# Effect of Locally Manufactured Niger Seed Oil on Lipid Profile Compared to Imported Palm and Sunflower Oils on Rat Models

**DOI:** 10.1155/2018/7846350

**Published:** 2018-05-02

**Authors:** Zewdie Mekonnen, Abrha Gebreselema, Yohannes Abere

**Affiliations:** ^1^Department of Biochemistry, College of Medicine and Health Sciences, Bahir Dar University, Bahir Dar, Ethiopia; ^2^Department of Biomedical Research, Biotechnology Research Institute, Bahir Dar University, Bahir Dar, Ethiopia

## Abstract

**Background:**

Different types of dietary lipids have been shown to affect lipid metabolism and lipid profile differently.

**Objective:**

This study aims to assess the effect of local niger seed oil on serum lipid profile compared to palm oil and sunflower oil in rats.

**Methods:**

The effect of the 15% plant oils on serum lipid profile, body weight gain percentage, and feed efficiency ratio was assessed after 8 weeks of experimental period.

**Results and Conclusion:**

The 15% niger seed oil showed decrease and increase in the level of lipid profile as compared to rats fed with 15% palm oil and sunflower oil (except Triacylglycerol), respectively. The 15% niger seed oil showed significant decrease and increase in body weight gain percentage as compared to the 15% palm oil and 15% sunflower oil, respectively. The feed efficiency ratio was significantly higher and lower in the 15% niger seed oil compared to rats fed with 15% sunflower oil and control group and the palm oil fed rats, respectively. The current study concluded that consumption of locally manufactured niger seed oil decreased the blood lipid profiles, body weight gain percentage, and feed efficiency ratio as compared to palm oil. Utilization of oils containing more unsaturated fatty acids like niger seed oil is recommended to reduce the risk of developing cardiovascular disease.

## 1. Introduction

Lipoprotein disorder is among the most common metabolic diseases occurring in human. It may lead to coronary heart disease [1–3]. Cardiovascular diseases are a group of diseases of the heart and blood vessels which include coronary heart disease, cerebrovascular disease, peripheral arterial disease, rheumatic heart disease, congenital heart disease, deep vein thrombosis, and also pulmonary embolism. They are reported to be responsible for 31% of global deaths [4]. Coronary heart disease develops from the occlusion of coronary vessels by atherosclerotic plaques [5]. Excess levels of blood cholesterol accelerate atherogenesis. Controlling the blood cholesterol level reduces the incidence of coronary heart disease [6, 7]. Knowledge about the levels of cholesterol subfractions is reported to be more meaningful than simple plasma cholesterol level. The higher the level of LDL-c, the greater the risk of atherosclerotic heart disease. Conversely, the higher the level of HDL-c, the lower the risk of coronary heart disease [8]. Different types of dietary lipids have been reported to affect lipid metabolism and serum lipid profile differently [9]. Plasma cholesterol levels are moderately decreased when low-cholesterol diets are used [10, 11]. It is now generally believed that vegetable oils decrease plasma cholesterol levels, although they differ in their cholesterol-lowering capacity. Furthermore, the effect of dietary cholesterol on plasma cholesterol levels may be influenced by the types of fatty acid consumed [12–15].

Oilseeds are reported as mainstay of rural and national economy in Ethiopia. Niger is an oilseed crop mainly cultivated in different parts of Ethiopia and India [16]. Niger seed oil provides about 50–60% of the oil for domestic consumption in Ethiopia [17]. It is also used as an oilseed crop in India, where it provides about 3% of the edible oil requirement of the country [18]. Palm oil, obtained from the fruits of the palm trees, is the most widely produced edible vegetable oil in the world, used in food preparation for over 5,000 years, and its nutritional and health attributes have been well documented [19, 20]. The oil is consumed in its fresh state or at various levels of oxidation. Feeding experiments in various animal species and humans have highlighted controversial evidences on the beneficial and harmful effects of fresh palm oil on health. The reported benefits include reduction in the risk of arterial thrombosis and atherosclerosis, inhibition of cholesterol biosynthesis, platelet aggregation, and reduction in blood pressure [21]. On the other hand, when used in the oxidized state, it possesses potential danger to the physiological and biochemical functions of the body. The reduction of the dietetic level of oxidized oil or the level of oxidation may reduce the health risk [21]. Sunflower oil is one of the major vegetable oils produced worldwide. Sunflower plants can only be grown in limited geographical locations because of the soil and climatic conditions required [22]. The oil of sunflower contains more polyunsaturated fatty acids and many other molecules that are responsible for the different health benefits of sunflower oil. The health effect assessment of sunflower oil was carried out in the 1970s and 1980s and was essential to our understanding of the specific effects of different fatty acids on heart health. Due to its high omega-6 fatty acid, sunflower oil is reported to improve blood lipid profile and reduce the risk of cardiovascular disease. The oil is used in spread manufacture, in cooking, and for dressing salads [23]. From many years of research, it has been established that the primary cholesterol-elevating fatty acids are the saturated fatty acids with 12 (lauric acid), 14 (myristic acid), and 16 carbon atoms (palmitic acid) with a concomitant increase in the risk of coronary heart disease. World Health Organization in its report in 2005 stated that there is a convincing evidence that palmitic oil consumption contributes to an increased risk of developing cardiovascular diseases [2, 3, 19, 24].

The total cholesterol, LDL-c, HDL-c, and TAG are collectively called blood lipids. Their levels could be modified by the type and amount of fat in the diet [25, 26]. The comparison of oils from different origin could be useful to establish the quality of oil and give direction or clue about the impact of their consumption on the nutritional state, cardiovascular disease, and related health of consumers. As to the researchers' knowledge, there was no literature or studies conducted in Ethiopia which can indicate the effect of niger seed oil on serum lipid profile compared to the imported palm oil and sunflower oil. Therefore, this study attempts to compare the effect of niger seed oil manufactured in Bahir Dar to the imported palm and sunflower oils on serum lipid profile in rat model.

## 2. Materials and Methods

### 2.1. Oil Sample Collection

Niger seed oil was purchased from niger seed oil extracting local factory in Bahir Dar, Ethiopia, and imported palm and sunflower oils were purchased from a supermarket in Bahir Dar, Ethiopia.

### 2.2. Experimental Diets Preparation

Test diets were prepared by mixing sample oils with normal commercial rat pellet to contain 15% of the oils. Each of the 15% test diets was prepared by adding 15 g niger seed oil, 15 g palm oil, and 15 g sunflower oil to 85 g rat pellet in a separate container. The oil and the rat pellet were mixed manually and left to absorb the vegetable oils at room temperature overnight and stored at 20°C before the feeding trial is conducted.

### 2.3. Experimental Animals

Male Wistar albino rats (*n* = 12) were purchased from Animal Science Department, Gondar University, Gondar, Ethiopia. All animals were given one week of acclimatization in animal housing conditions before being used for the study. All the animals were fed with standard animal feed and had access to water and were handled in accordance with the National Institutes of Health (NIH) Guidelines for Care and Use of Laboratory Animals. The rats were divided into 4 groups, each containing 3 rats. Each group was housed in a separate cage. Group 1 served as the normal control and received standard animal feed and water; group 2 was fed with 15% niger seed oil; group 3 was fed with 15% of sunflower oil; and group 4 was fed with 15% palm oil. The experiment lasted for 8 weeks.

### 2.4. Biological Evaluation

During the experimental period, food intake (FI) was recorded every second day for each group, and the animals were weighted twice weekly in all groups. The biological values of different diets were assessed by the determination of body weight gain percentage (BWG %) which was calculated at the end of the experimental period, using the following equation:

(1) body  weight  gain  percentageBWG%=Final  b.wt−Initial  b.wt×100Initial  b.wt,

where, b.wt is body weight of rat.

The feed efficiency ratio (FER) was calculated using the following equation [27]:

(2) feed  efficiency  ratioFER=gain  in  body  weightgfeed  consumedg.

### 2.5. Blood Collection and Serum Separation

At the end of the experiment (8 weeks), the feeding of rats was stopped for 12 hours before they were scarified by cervical dislocation and blood was taken through cardiac puncture by trained individual. The serum was separated through centrifugation with speed of 3000 revolutions per minute at room temperature for 10 minutes. Then, test tubes were placed in ice-box and transported to the GAMBY General Teaching Hospital for the analysis.

### 2.6. Laboratory Analysis

The serum levels of total cholesterol, triglyceride, HDL-c, and LDL-c were analyzed by ABX Pentra 400 clinical chemistry autoanalyzer (manufactured by HORIBA ABX SAS) as per the manufacturer's instructions. To ensure the accuracy and precision of the test results, all preanalytical, analytical, and postanalytical precautions were taken into consideration. The results obtained from the laboratory staff were validated and verified by trained personnel before release. In addition, to maintain internal quality control, known standards were run and the equipment was calibrated prior to analysis. As external quality control, the laboratory also participates in the international digital Proficiency Testing (PT) program.

### 2.7. Statistical Analysis

All statistical calculations were performed on the Statistical Package for Social Sciences (SPSS) version 20.0 software (IBM Corp., released in 2011, Armonk, NY). All data were expressed as the mean ± SD. Independent sample *t*-test and one-way ANOVA were used. In all the statistical tests, a confidence level of 95% and *p* < 0.05 were considered significant.

## 3. Result

**Figure 1:**
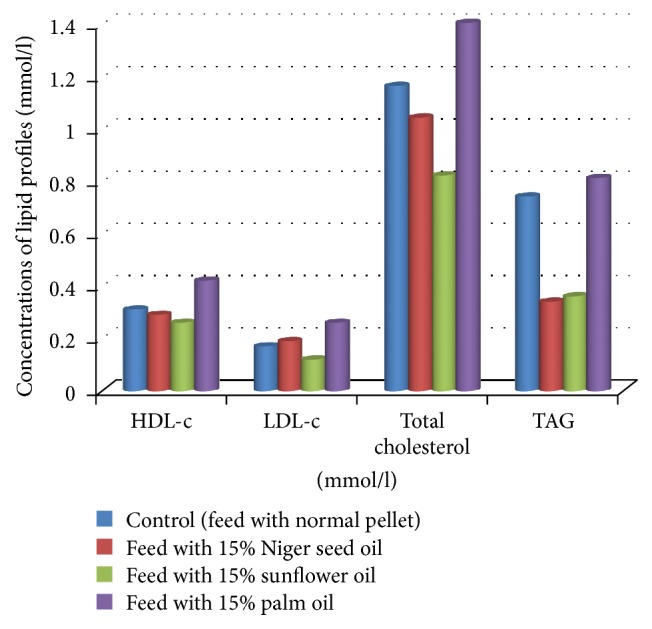
The comparisons of the mean values of total cholesterol, TAG, HDL-c, and LDL-c of blood serum at the end of study period.

The current study showed decrease in the level of total cholesterol, triglyceride, and HDL-c and slight increase in the level of LDL-c in rats fed with 15% niger seed oil as compared to the control group. It also showed increase in the level of total cholesterol, HDL-c, and LDL-c and slight decrease in the level of triglyceride in rats fed with 15% niger seed oil compared to rats fed with 15% sunflower oil. High levels of total cholesterol, HDL-c, LDL-c, and TAG were observed in rats fed with 15% palm oil compared to all other groups (Table 1 and Figure 1).

**Figure 2:**
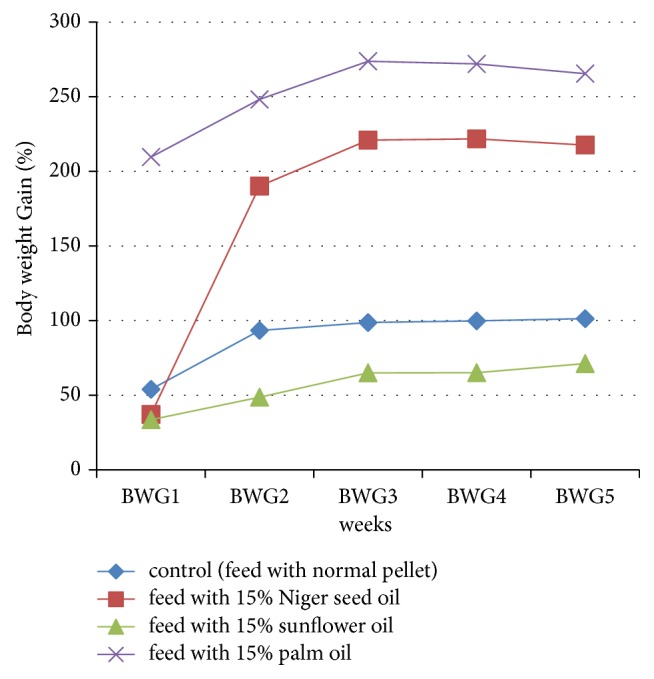
Characteristics of the body weight gain percentage (BWG %) of experimental animals (Wistar albino rats) (BWG1: body weight gain percentage of rats in the first one week; BWG2: body weight gain percentage of rats in the 2nd two weeks; BWG3: body weight gain percentage of rats in the 3rd two weeks; BWG4: body weight gain percentage of rats in the 4th two weeks; BWG5: body weight gain percentage of rats in the final 8th weeks).

As shown in Table 2 and Figure 2, significant (*p* < 0.05) decrease in body weight gain percentage was observed in rats fed with 15% niger seed oil compared with rats fed with 15% palm oil and significant (*p* < 0.05), as well as increase in body weight gain percentage compared to control group. A significant (*p* < 0.05) decrease in the body weight gain percentage in rats fed with 15% sunflower oil compared to rats fed with 15% niger seed oil and 15% palm oil and control group was shown.

**Figure 3:**
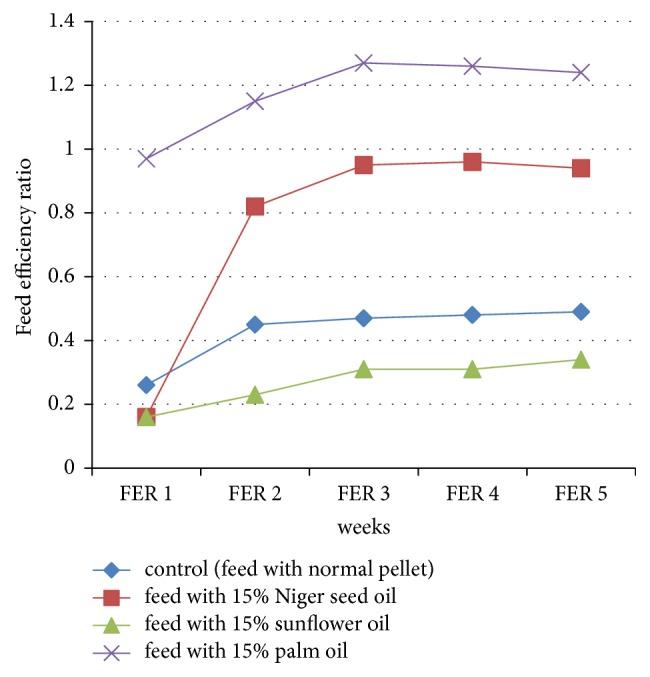
The characteristics of feed efficiency ratio (FER) of experimental animals (Wistar albino rats) (FER1: FER of rats in the first one week; FER2: FER of rats in the 2nd two weeks; FER3: FER of rats in the 3rd two weeks; FER4**: **FER of rats in the 4th two weeks; FER5: FER of rats in the final 8th weeks).

The study also showed a significant (*p* < 0.05) increase in the feed efficiency ratio (FER) of rats fed with 15% niger seed oil compared to the control group throughout the study period. It also indicated nonsignificant (*p* > 0.05) increase in feed efficiency ratio (FER) of rats fed with 15% niger seed oil compared to rats fed with 15% sunflower oil in the first week of the experimental period and significant (*p* < 0.05) increase was observed after the first week of the experimental period. Rats fed with palm oil had a significant (*p* < 0.05) increase in feed efficiency ratio (FER) as compared to other groups (Table 3 and Figure 3).

## 4. Discussion

The oil content of niger is reported to be 42–44% [28–30]. It has been reported to contain up to 20.9% carbohydrate and 27.8% protein [16]. Niger seed oil has a fatty acid composition typical for seed oils of the Asteraceae plant family. The oil extracted from niger seed cultivated in Ethiopia was reported to contain more than 70% linoleic acid [16, 28–31], while the one cultivated in India contains approximately 55% linoleic acid [16]. In all other works done so far on the fatty acid composition of niger, linoleic acid is unequivocally the dominant fatty acid present in niger seed oil followed by palmitic, oleic, and stearic acids [28–32]. Regarding the fatty acid profile, niger seed oil resembles that of sunflower oil with its high content of linoleic acid, which may be up to 85% depending on the origin [17]. Due to its high linoleic acid content, the oil of niger seed is reported to be nutritionally important. The niger seed oil is also reported to have significant antioxidant activity due to its content of sterols and tocopherols [16].

The palm oil has been reported to have 50% saturated fatty acids esterified with glycerol. The saturated fatty acid content of palm oil included palmitic acid (C-16:0), and the unsaturated fatty acid content included 10% linoleic acid (C-18:2), which is omega-6 fatty acid. Palm oil also contains vitamin K, magnesium, tocotrienols, and small amounts of squalene and ubiquinone [33]. In addition to the polyunsaturated fatty acids, sunflower oil also contains a number of other compounds including tocopherols, plant sterol and stanol esters, phospholipids, carotenoids, and trace elements. It contains alpha-tocopherol, which makes it resistant to photooxidation, and it is low in gamma-tocopherol, which is required to provide stability against oxidation [23].

The levels of total cholesterol and LDL-c were significantly (*p* < 0.05) higher in rats fed with niger seed oil as compared to rats fed with sunflower oil. The mean values of total cholesterol and TAG were significantly (*p* < 0.05) higher in rats feed with niger seed oil compared to control group. The difference in the mean value of TAG between niger seed oil and sunflower oil was not significant (*p* > 0.05). There was also nonsignificant (*p* > 0.05) increase in the level of HDL-c in rats fed with niger seed oil as compared to rats fed with sunflower oil. These observed results might be due to the high percentage of polyunsaturated fatty acid in sunflower oil compared to niger seed oil [21, 34]. Polyunsaturated fatty acids have been reported to facilitate the transportation and utilization of lipids [34]. Studies reported that sunflower oil is very high in polyunsaturated fatty acid and low in saturated fatty acid contents. They reported that standard sunflower oil is predominantly composed of linoleic acid (C-18:2) and oleic acid (C-18:1). These two acids account for about 90% of the total fatty acid content of sunflower oil. The remaining 8–10% is comprised of palmitic acid (C-16:0) and stearic acid (C-18:0) [23]. Slight increase in the level of triglyceride has been shown in rats fed with 15% sunflower oil as compared to rats fed with niger seed oil. This result agreed with the finding of a study that reported that increasing the polyunsaturated fatty acid content in food increases the Triacylglycerol level [35, 36]. The levels of total cholesterol, HDL-c, LDL-c, and TAG were significantly (*p* < 0.05) higher in rats fed with 15% palm oil compared to control group, rats fed with 15% niger seed oil, and rats fed with 15% sunflower oil.

The increase in the level of lipid profiles in rats fed with palm oil compared to the other groups might be due to the percentage of saturated fatty acids. The reported percentage of saturation and unsaturation in the fatty acid composition of palm oil was one to one, where as in niger seed and sunflower oil, the percentage of saturation was relatively lower compared to palm oil. The existence of more unsaturated fatty acid percentage in niger seed and sunflower oil might help the experimental animal to process the feed more efficiently compared to the palm oil-fed rats [21]. High intake of saturated fatty acid has been linked to increased cholesterol levels which can lead to cardiovascular disease [37]. In line with this, dietary recommendations have limited the intake of saturated fatty acids for the prevention of cardiovascular disease. World Health Organization's 2005 report stated that there is a convincing evidence that palm oil consumption contributes to an increased risk of developing cardiovascular disease [24]. In line with World Health Organization's 2005 report, a number of studies suggested the association of high contents of saturated fats in palm oil with the detrimental atherogenic profile [23, 38, 39]. Other studies done on the effect of palm oil on plasma lipoprotein profile reported positive correlation with the risk of developing cardiovascular disease [15, 40]. A study carried out for determination of the effect of lipid profile by supplementing polyunsaturated fatty acids like linoleic acid and saturated fatty acids like palmitic acid and stearic acid also reported similar results [41]. Another research done by supplementation of dietary fat containing more unsaturated fatty acid (saturated fatty acids replaced with polyunsaturated fatty acids) reported more LDL fractional catabolic rate that might contribute to variability of plasma cholesterol levels [42].

The significant decrease in body weight gain percentage of rats fed with 15% sunflower oil as compared to rats fed with 15% niger seed oil, rats fed with 15% palm oil, and control group and the significant (*p* < 0.05) decrease in body weight gain percentage of rats fed with 15% niger seed oil compared with rats fed with 15% palm oil might be because of the fatty acid composition difference of sunflower oil, niger seed oil, and palm oil [28–32]. Researchers indicated that the utilization of polyunsaturated fatty acids increases the utilization of proteins and cholesterol and might assist in losing weight [35, 36]. Research done on dietary fatty acid with utilization of polyunsaturated fatty acids reported that the polyunsaturated fatty acids facilitated the lipid metabolism and resulted in decrease in obesity [43, 44]. The rats fed with palm oil have shown high body weight gain percentage compared to other groups. This might be because of fatty acid composition differences in which the palm oil contains more saturated fatty acid compared to the fatty acid composition of sunflower oil and niger seed oil; particularly the palm oil contains more palmitic acid [40, 45]. Different studies reported that saturated fatty acids (SFAs) have been shown to produce higher rates of weight gain compared with other types of fatty acids [36, 42, 43, 46].

The significant increase in feed efficiency ratio (FER) in rats fed with niger seed oil compared to rats fed with sunflower oil and control group and significant decrease compared to rats fed with palm oil (Table 3 and Figure 3) might be due to the fatty acid composition difference of the oils which precipitated to either accumulation of the body lipid or more processing of the body lipid. In the current study, the rats fed with palm oil gained more progressive body weight compared to the other groups. This might contribute to the significant increase in body weight gain percentage and feed efficiency ratio in rats fed with palm oil as compared to the other groups. Different research findings indicated that more lipid accumulation affects the body weight gain percentage and the feed efficiency ratio [35, 36, 43, 44]. Other research findings also reported that the utilization of unsaturated fatty acids such as monounsaturated fatty acids reduced fat gain, which agreed with the current study, in which the utilization of more unsaturated fatty acid containing niger seed oil and sunflower oil showed significant decrease in body weight gain percentage and feed efficiency ratio compared to palm oil [9, 36, 46].

## 5. Conclusion

The current study concluded that consumption of locally manufactured niger seed oil decreased the blood lipid profiles, body weight gain percentage, and feed efficiency ratio as compared to palm oil. It also indicated decease in blood lipid profile, body weight gain percentage, and feed efficiency ratio in rats fed with sunflower oil as compared to rats fed with palm oil and niger seed oil. Niger seed oil lowers the plasma lipid profile that precipitated to low risk of cardiovascular disease. Because the polyunsaturated fatty acids have been found to facilitate lipid transportation and metabolism, utilization of oils containing more unsaturated fatty acids like niger seed oil and sunflower oil is recommended to reduce the risk of developing cardiovascular diseases.

## Figures and Tables

**Table 1:** The effect of niger seed oil, sunflower oil, and palm oil on blood serum total cholesterol, TAG, HDL-c, and LDL-c at the end of study period.

Group	HDL-c (mmol/l)	LDL-c (mmol/l)	Total cholesterol (mmol/l)	TAG (mmol/l)
Control (feed with normal pellet)	0.31 ± 0.01^a^	0.17 ± 0.01^a^	1.16 ± 0.02^a^	0.74 ± 0.0.01^a^
Feed with 15% niger seed oil	0.29 ± 0.03^a^	0.19 ± 0.02^a^	1.04 ± 0.03^b^	0.34 ± 0.01^b^
Feed with 15% sunflower oil	0.26 ± 0.02^a^	0.12 ± 0.01^ab^	0.82 ± 0.02^ab^	0.36 ± 0.02^b^
Feed with 15% palm oil	0.42 ± 0.03^b^	0.26 ± 0.03^c^	1.4 ± 0.003^c^	0.81 ± 0.02^c^

Data were expressed as means ± SD (*p* < 0.05). Values with different letters within the same column are statistically significant.

**Table 2:** The characteristic mean level of the body weight gain percentage (BWG %) of experimental animals (Wistar albino rats) through different weeks of the experimental period.

Feed	Body weight gain % of rat in the first one week	Body weight gain % of rat in the 2nd two weeks	Body weight gain % of rat in the 3rd two weeks	Body weight gain % of rat in the 4th two weeks	Body weight gain % of rat in the final 8th weeks
Control (feed with normal pellet)	53.96 ± 0.94^a^	93.45 ± 0.96^a^	98.74 ± 0.42^a^	99.83 ± 0.43^a^	101.34 ± 0.87^a^
Feed with 15% niger seed oil	37.15 ± 0.97^b^	190.13 ± 0.70^b^	220.93 ± 0.65^b^	221.77 ± 0.67^b^	217.65 ± 0.51^b^
Feed with 15% sunflower oil	33.62 ± 1.4^c^	48.68 ± 1.15^c^	65.04 ± 0.87^c^	65.11 ± 0.90^c^	71.23 ± 0.96^c^
Feed with 15% palm oil	209.57 ± 0.51^d^	248.20 ± 1.0^d^	273.73 ± 0.38^d^	271.97 ± 0.23^d^	265.47 ± 0.47^d^

Data were expressed as means ± SD (*p* < 0.05). Values with different letters within the same column are statistically significant.

**Table 3:** The characteristic mean level of feed efficiency ratio (FER) of experimental animals (Wistar albino rats) through different weeks of the experimental period.

Feed	FER of rat in the first one week	FER of rat in the 2nd two weeks	FER of rat in the 3rd two weeks	FER of rat in the 4th two weeks	FER of rat in the final 8th weeks
Control (feed with normal pellet)	0.26 ± 0.01^a^	0.45 ± 0.01^a^	0.47 ± 0.006^a^	0.48 ± 0.01^a^	0.49 ± 0.006^a^
Feed with 15% niger seed oil	0.16 ± 0.02^b^	0.82 ± 0.044^b^	0.95 ± 0.064^b^	0.96 ± 0.067^b^	0.94 ± 0.067^b^
Feed with 15% sunflower oil	0.16 ± 0.035^b^	0.23 ± 0.055^c^	0.31 ± 0.056^c^	0.31 ± 0.056^c^	0.34 ± 0.069^c^
Feed with 15% palm oil	0.97 ± 0.015^c^	1.15 ± 0.012^d^	1.27 ± 0.006^d^	1.26 ± 0.012^d^	1.24 ± 0.032^d^

Data were expressed as means ± SD (*p* < 0.05). Values with different letters within the same column are statistically significant. In all comparisons the *p*-value is less than 0.05 except the comparison between 15% Sunflower oil and 15% Niger seed oil on FER of Rat in the first one week of the experiment in which the *p* > 0.05.

## Data Availability

All relevant data are included with the manuscript.

## Abbreviations

BWG %: Body weight gain percentage
CHD: Coronary heart disease
CVD: Cardiovascular disease
FER: Feed efficiency ratio
FI: Food intake
T-C: Total cholesterol
HDL-c: High-density lipoprotein cholesterol
LDL-c: Low-density lipoprotein cholesterol
TAG: Triacylglycerol
NSO: Niger seed oil
SFO: Sunflower oil
PO: Palm oil
PUSFA: Polyunsaturated fatty acid
LA: Linoleic acid
SD: Standard deviation
SFA: Saturated fatty acids
mmol/l: Millimoles per liter.
